# Editorial: Natural antioxidants and cardiovascular diseases: a pathway to prevention

**DOI:** 10.3389/fcvm.2026.1813976

**Published:** 2026-03-11

**Authors:** Roberta Giordo, Anna Maria Posadino

**Affiliations:** 1 Department of Human Science for Promotion of Quality of Life, University San Raffaele, Rome, Italy; 2 Department of Biomedical Sciences, Faculty of Medicine and Surgery, University of Sassari, Sassari, Italy

**Keywords:** cardiovascular diseases - complications, endogenous antioxidant, endothelial dysfunction, nature-derived compounds, oxidative stress

Cardiovascular diseases (CVDs) continue to represent the leading cause of morbidity and mortality worldwide, despite remarkable advances in pharmacological and interventional therapies. This persistent burden underscores the need for complementary and preventive strategies that address upstream mechanisms driving cardiovascular pathology. Among these, oxidative stress has emerged as a central, unifying process linking endothelial dysfunction, inflammation, metabolic dysregulation, and myocardial injury. In this context, natural antioxidants have gained increasing attention due to their pleiotropic biological activities, generally favorable safety profiles, and potential suitability for long-term preventive approaches.

The Research Topic “*Natural Antioxidants and Cardiovascular Diseases: A Pathway to Prevention”* brings together five complementary contributions that collectively advance our understanding of how endogenous antioxidant systems and nature-derived compounds may influence redox balance, inflammatory signaling, lipid metabolism, vascular function, and mitochondrial homeostasis across diverse cardiovascular conditions. Rather than focusing on a single molecule or disease entity, this Research Topic highlights convergent biological pathways through which distinct classes of natural antioxidants may exert cardioprotective effects.

An important contribution explores the relationship between endogenous antioxidant regulation and acute inflammatory cardiac injury. The study examining melatonin deficiency in acute myocarditis provides clinical evidence linking reduced nocturnal melatonin secretion, assessed via urinary 6-sulfatoxymelatonin levels, with the presence of myocardial inflammation Chen et al. These findings support the concept that impaired chronobiological and redox homeostasis may accompany acute cardiac injury. At the same time, the observational design and reliance on a single surrogate biomarker highlight the need for cautious interpretation and motivate future studies incorporating longitudinal sampling and more comprehensive circadian phenotyping. Beyond its direct antioxidant capacity, melatonin is increasingly recognized as a modulator of mitochondrial function and immune responses, positioning it at the intersection of oxidative stress and inflammatory cardiomyopathy.

Atherosclerosis, the pathological substrate underlying most cardiovascular events, is addressed from complementary mechanistic perspectives. One review focuses on the crosstalk between lipid metabolism and macrophage biology within the atherosclerotic plaque, illustrating how natural products may modulate foam cell formation, macrophage polarization, and inflammatory signaling Qian et al. By integrating evidence from experimental models, the review reinforces the concept that antioxidant effects in the vascular wall are inseparable from immunometabolic regulation. While much of this literature remains preclinical and heterogeneous in terms of compound formulations, dosing paradigms, and endpoints, the synthesis highlights biologically coherent pathways that can inform hypothesis-driven translational development.

From a population-based perspective, the association between dietary flavonoid intake and hypertension is examined using NHANES data Niu et al. The reported inverse association between total flavonoid consumption, particularly anthocyanidins and flavan-3-ols, and hypertension prevalence provides epidemiological support for a potential role of flavonoid-rich dietary patterns in cardiovascular prevention. Notably, the observed effect modification by age and metabolic status suggests that inter-individual context may shape responsiveness, reinforcing the rationale for more tailored nutritional approaches. Consistent with the nature of cross-sectional population analyses, these findings should be interpreted as associations rather than evidence of causality, and dietary exposure estimates, derived from self-reported intake, may be influenced by measurement variability and residual confounding. Nevertheless, the large, heterogeneous sample and the real-world setting strengthen the translational relevance of the observations, providing an epidemiological counterpart to experimental and mechanistic studies and helping prioritize hypotheses for prospective validation and intervention trials.

Another review examines the therapeutic potential of natural organosulfur compounds, particularly those derived from garlic and related plant sources, in atherosclerosis Tang et al. This contribution highlights multi-target actions involving lipid metabolism, endothelial protection, inflammatory pathway modulation, and activation of antioxidant defense systems such as the Nrf2/ARE axis. Importantly, the review also underscores practical translational challenges, including variable bioavailability and a lack of standardized formulations, that can contribute to inconsistent efficacy signals across studies and represent key priorities for clinical development.

The cardioprotective potential of complex polyphenolic mixtures is further illustrated by the experimental study on Yellow Wine Polyphenolic Compounds in myocardial ischemia–reperfusion injury Xu et al. Using an *in vivo* rat model, the study demonstrates reduced infarct size, preserved cardiac function, and improved mitochondrial dynamics via Nrf2-dependent signaling. While extrapolation to human disease requires careful consideration of species differences, dosing, and timing relative to clinical ischemic events, the work strengthens the mechanistic link between redox-sensitive programs and mitochondrial quality control as actionable nodes in acute cardiac injury.

Taken together, the contributions in this Research Topic point to a common message: oxidative stress in cardiovascular disease does not act in isolation. Instead, it is closely intertwined with inflammation, metabolic regulation, immune responses, and mitochondrial function. In this complex biological context, natural antioxidants, whether produced endogenously or introduced through diet, tend to exert their effects through coordinated, multi-target actions. These effects often involve the activation of cellular stress-response pathways, such as Nrf2 signaling, and broader immunometabolic adaptations, rather than simple direct neutralization of reactive oxygen species.

The potential clinical impact of these compounds will therefore depend not only on their biological plausibility, but also on the strength of the supporting clinical evidence. Progress in this field will require standardized and well-characterized formulations, reliable biomarkers to assess exposure and biological response, and strategies to identify the patient groups most likely to benefit.

Looking ahead, this Research Topic also highlights clear priorities for future research. These include rigorously designed clinical trials, improved standardization of complex natural preparations, and mechanistic studies that integrate redox biology with immune and metabolic pathways using clinically relevant models. By encouraging dialogue across basic, translational, and population-based research, this collection aims to support the rational development of nature-derived strategies as part of preventive and personalized approaches to cardiovascular disease.

In conclusion, “*Natural Antioxidants and Cardiovascular Diseases: A Pathway to Prevention”* offers an integrative perspective on how redox-modulating strategies derived from nature may complement existing cardiovascular therapies. By bridging experimental, epidemiological, and clinical insights, while maintaining an appropriately cautious view of current evidence, this Research Topic provides a balanced framework for advancing antioxidant-based approaches within preventive and personalized cardiovascular medicine.

